# Role of Water in Low‐Temperature CO_2_ Reduction at Defect‐Rich TiO_2_

**DOI:** 10.1002/anie.7574479

**Published:** 2026-05-13

**Authors:** Justin Klimek, Filip Hallböök, Niko Kruse, Fangliang Li, Sara Blomberg, Katharina Al‐Shamery, Lars Mohrhusen

**Affiliations:** ^1^ Institute of Chemistry Carl von Ossietzky University of Oldenburg Oldenburg Germany; ^2^ Department of Process and Life Science Engineering Lund University Lund Sweden

**Keywords:** C1 building blocks, CO_2_ activation, defect chemistry, heterogeneous catalysis, near‐ambient pressure X‐ray photoelectron spectroscopy, titanium dioxide

## Abstract

Point defects in titanium dioxide (TiO_2_) are the most relevant reaction sites for the activation of oxygenates. Herein, we probe the activation of CO_2_ on highly defective TiO_2_ in situ using synchrotron‐based near‐ambient pressure X‐ray photoelectron spectroscopy between 0.1 mbar and 2.6 mbar from room temperature to 700 K. Multiple carbon surface intermediates form upon CO_2_ activation. The presence of key intermediates acts as a fingerprint for the population of different reaction pathways, that is, promotion or suppression of selected reaction steps in various gas environments. In the absence of potent H/OH donors, the formyl, glyoxal, formaldehyde and carbene pathways are populated simultaneously. However, aqueous atmospheres boost intermediate formation and promote oxygen‐rich organic molecules, suppressing coke formation along the carbene pathway. Thermal loss of hydroxyls above ≈550 K triggers the population of such oxygen‐lean routes, along with a decrease in carbon intermediates, converging to the chemistry under water‐free conditions. Our results highlight reduced titania as a noble metal‐free CO_2_ activation catalyst and demonstrate how water can be used to favor the desired product distribution. The findings herein will guide the development of sustainable catalysts from heavily reduced oxides, for example, black titania, for platform chemicals based on CO_2_ as a building block.

## Graphical abstract

Our results highlight the potential of reduced titania as a noble metal‐free CO_2_ activation catalyst and show the activation of CO_2_ on highly defective TiO_2_ using in situ synchrotron‐based NAP‐XPS from room temperature to 700 K. The distribution of surface intermediates can be decoded as a simultaneous population of reaction routes, revealing water (OH) as a potent hydrogen donor to steer between oxygen‐lean and oxygen‐rich reaction pathways.

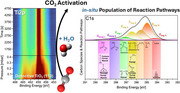

## 1 Introduction

To reduce atmospheric CO_2_ concentrations [1] and handling the increasing energy demand [2] necessitate new carbon utilization technologies (CCU) to fabricate platform chemicals from CO_2_, for example, from industrial point sources [3]. Even though there is a diverse choice of (heterogeneous) catalysts for the formation of C_1_ and C_2_ products from CO_2_, they often rely on critical raw materials such as (noble) transition metals [4, 5] or energy‐intensive reaction conditions like high pressures or temperatures [6]. Even in artificial photosynthesis, critical materials like gold, platinum, or silver are often used as reactive sites or light absorbers in nanostructured catalysts [7].

Because of environmental effects [8], abundance and therefore price, as well as (photo‐)catalytic properties, we consider an alternative catalyst, that is, defective TiO_2_ as an optimal model system to study CO_2_ reduction [9]. Titania has been investigated by several groups under the aspect of the surface chemistry of oxygenates, including CO_2_ predominantly on rutile TiO_2_(110) [10, 11, 12, 13, 14]. Under ultra‐high vacuum (UHV) conditions, CO_2_ adsorbs weakly on the TiO_2_(110) surface. While CO_2_ adsorbed on the Lewis‐acidic Ti^4+^ centers already desorbs around 137 K, CO_2_ binds stronger to oxygen vacancies, desorbing at 166 K [15, 16]. Low‐temperature scanning tunneling microscopy (STM) supports that oxygen vacancies are preferred adsorption sites due to their excess electrons that give them a base‐like character [17]. Infrared spectroscopy also shows preferential binding to oxygen vacancies before binding to titanium centers or even horizontally at bridging oxygens, the latter allowing for carbonate formation [18, 19]. Temperature programmed desorption (TPD) and infrared spectroscopy studies suggest that CO_2_ is displaced by water at temperatures above 75 K due to the weak binding. However, a bicarbonate can be formed when both molecules are dosed simultaneously [15, 20].

The outstanding relevance of defects in titania does not only apply to the adsorption of CO_2_ but also to the activation of more reactive oxygenates like methanol or acetone in UHV [21, 22]. The base‐like properties of oxygen vacancies allow for dissociation of oxygenates like alcohols under the formation of alkoxy species. These can undergo C–O cleavage or dehydrogenation en route to, for example, selective partial oxidation or coupling reactions [23, 24].

Beyond UHV conditions, more recent studies have focused on CO_2_ activation on TiO_2_ under near ambient pressure (NAP) conditions at room temperature. A NAP‐STM study showed that CO_2_ on rutile TiO_2_(110) forms an ordered structure of weakly bound CO_2_ at pressures of 0.7 mbar [25]. A combined study using NAP‐STM, NAP‐X ‐ray photoelectron spectroscopy (NAP‐XPS), and density functional theory (DFT) demonstrated that CO_2_ heals defects in vacuum‐annealed TiO_2_(110) surfaces and fosters diffusion of oxygen vacancies along the surface [26]. Here, the NAP‐STM allowed for investigation of the vacancy diffusion, showing both linear and bridged CO_2_‐species. The samples used herein are more strongly reduced than in any literature on CO_2_ exposures on defective TiO_2_ in the mbar regime. Those reported Ti^3+^ contents of 5%–20%, but with no clear Ti^2+^ shoulder visible [25, 26]. The initial defect concentrations from Kim's work [26] (using a photon energy of 590 eV) somewhat correspond to the defect concentration in our sample after oxidation. This emphasizes the need to investigate the role of defects under operando conditions to clarify the remaining question of what happens to CO_2_ after its initial activation. Foremost, the influence of hydrogen donors on the formation of C_1_ and C_2_ products and, in general, the impact of reaction conditions on the distribution of various reaction pathways, has not been discussed yet.

In this in situ NAP‐XPS study, we report the activation of CO_2_ on strongly defective rutile TiO_2_ for the first time, accompanied by surface oxidation, and shed light on the pressure dependence. We demonstrate the crucial role of water, forming surface hydroxyls as a readily efficient hydrogen source for the population of different reaction pathways towards the formation of organic molecules. Our findings will guide the development of future applications of heavily reduced oxide materials like black titania [27] for the activation of CO_2_ and its utilization as a carbon building block for platform chemicals.

## 2 Results

### 2.1 Oxidation of Defective HR‐TiO_2_ Under CO_2_ Activation

In the following section, we discuss the oxidation of highly defective rutile TiO_2_(110) in near‐ambient pressure atmospheres of CO_2_, which accompanies the CO_2_ activation. These surfaces were prepared by argon ion bombardment (2 keV) of a rutile TiO_2_(110) single crystal at room temperature, which creates a rough, unordered, and oxygen‐deficient surface that is terminated mainly by Ti^3+^ and Ti^2+^. As we have shown in the past, no conventional low‐energy electron diffraction (LEED) pattern can be observed for such surfaces, indicating the absence of long‐range order [28]. The root mean square (RMS) roughness of the samples herein could not be determined due to lack of microscopy instrumentation. However, analog XPS and atomic force microscopy (AFM) studies of defective TiO_2_ samples exhibiting a similar defect composition created by comparable Ar^+^ ion bombardment showed a roughness of 0.42 nm [29, 30, 31]. Therefore, we assume a similar roughness herein. All samples in this work were prepared and investigated without atmospheric handling in a UHV preparation chamber and a directly connected NAP‐XPS setup at the HIPPIE beamline at the Max IV synchrotron laboratory in Lund, Sweden. Further details can be found in the supporting information.

**FIGURE 1:**
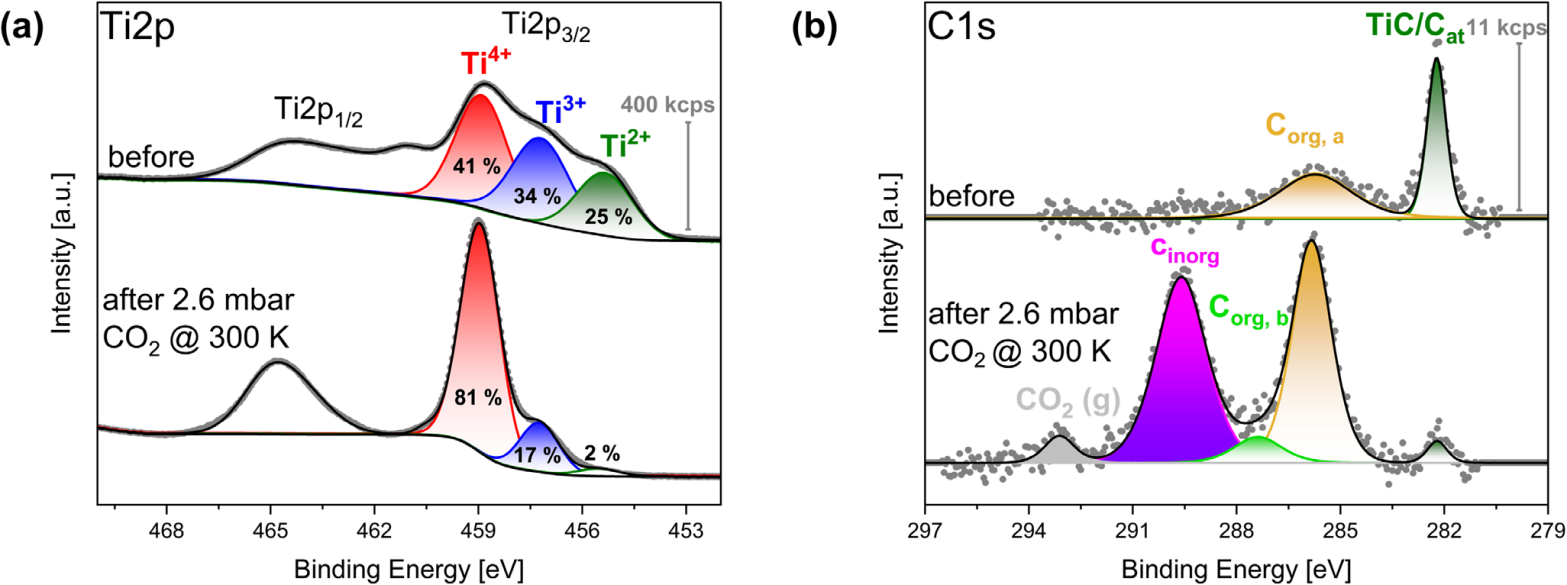
Ti2p (a) and C1s (b) AP‐XPS spectra of highly defective rutile TiO_2_(110) before (upper half) and after (lower half) exposure to 2.6 mbar of CO_2 (g)_ at room temperature_._ The measurements were conducted with 600 eV photon energy. Spectra before exposure were collected under UHV (∼10^−9^ mbar) conditions; spectra after exposure to 2.6 mbar for several minutes were collected after CO_2_ was pumped out and a pressure of ∼10^−5^ mbar was reached in the near‐ambient pressure cell. Scale bars apply for both spectra before and after exposure to CO_2_ in (a) or (b), respectively.

The highly defective TiO_2_ is readily reoxidized by 0.1–2.6 mbar pressures of CO_2_. The oxidation is evident from the Ti2p_3/2_ signal shown in Figure 1a. Each of the three chemical signals is split into two signals due to spin‐orbit coupling. Both signals provide equivalent information; hence, we will use the more intense Ti2p_3/2_ for further discussion and quantification. As the surface reduction was achieved by Ar^+^ ion bombardment, long‐range order is missing, and thus the signals are broadened compared to crystalline TiO_2_. Before the insertion of CO_2_, three chemical species of titanium in the form of three oxidation states (+IV, +III, and +II) can be identified in similar intensities. The first signal is the Ti^4+^ signal at 458.9 eV (in red). The second signal peaking at 457.2 eV (in blue) is assigned to the monoreduced Ti^3+^, while the last species is attributed to the higher reduced Ti^2+^ at 455.3 eV (in green). The signal positions were assigned according to literature, showing good agreement with earlier publications where Ti^4+^ was assigned to 458.8–459.2 eV, Ti^3+^ to 456.8–457.2 eV, and Ti^2+^ to 454.9–455.3 eV in UHV‐XPS [32, 33, 34]. After exposure to CO_2_ the Ti^4+^ signal becomes more pronounced, nearly doubling its relative integral area from 41 % to 81 % of the total Ti signal. A simultaneous decrease of both defect species can be observed, with the Ti^3+^ content being reduced by 50 % (from 34% to 17%), while Ti^2+^ is nearly completely oxidized, only making up 2% of the relative area, compared to the initially 25%. In total, 67% of the initial defect concentration is reoxidized. This matches the earlier studies by Hamlyn et al. [25] and Kim et al. [26] showing that relevant pressures of CO_2_ can remove oxygen vacancies and Ti^3+^ centers. The width of the Ti^3+^ signal decreases significantly after the oxidation, indicating some of the remaining defects might be located in defined interstitial sites in the bulk of the single crystal, which typically show smaller FWHMs.

After exposing the reduced surface to CO_2_, new carbon surface species appear on the TiO_2_ surface (Figure 1b). The two initially present species are a type of organic species at 285.8 eV (yellow), which was already present beforehand, possibly due to residual CO_2_ from prior measurements in the chamber. We assign this to not further‐specified organic compounds C_org,a_, as the exact nature of these signals remains unclear. The second signal at 282.2 eV (olive) is assigned to atomic carbon (C_at_) or a titanium carbide (TiC) formed during the Ar^+^ ion bombardment of the sample. Atomic carbon has been reported in the literature with C1s binding energies of ∼282.3 eV, matching our observations accurately. Alternatively, reports on the ion‐beam induced formation of TiC on Ti/C interfaces render an assignment to titanium carbide plausible [35]. C1s signals of such transition metal carbides are observed at slightly lower binding energies around 281.7 eV [35, 36, 37]. However, the relatively oxygen‐rich environment must be considered, possibly leading to upshifts in the signal positions.

After the CO_2_ exposure, three new species occur in the C1s spectra, as shown in Figure 1b. One signal arises from the gas phase CO_2_ at 293.0 eV from the decaying background pressure of the ambient pressure cell (confirmed with reference measurements, with the sample being retracted from the analyzer focus). We classify the second species at 289.6 eV as overlapping signals from oxidized inorganic compounds (CO_3_^2−^/HCO_3_^−^/CO) or formate, denoted C_inorg_, which will not be further deconvoluted due to finite resolution. Both carbonate species have been assigned similarly under NAP conditions on a Ag/TiO_2_ nanoparticle system in agreement with Raman spectroscopy [38] and the bicarbonate in UHV on a rutile TiO_2_(110) single crystal with XPS [39]. The typical UHV‐level XPS signal of CO adsorption on Anatase TiO_2_ is attributed to binding energies of ∼290 eV [40]. However, the assignment of this species as molecularly adsorbed CO_2_ (ad) is alternatively discussed in other NAP‐XPS studies of CO_2_ on TiO_2_ [25, 26]. The third species at 287.4 eV (green) is assigned as a second organic compound (C_org,b_), not further specified, with its peak position being similar to dioxoethylene species from ethanol adsorption [41]. In parallel a reduction in the TiC signal can be observed. Consumption of the carbide in a Boudouard‐type reaction may take place, converting CO_2_ and C to CO [42]. This could therefore indicate that carbon monoxide, as part of the C_inorg_ signal, is being formed, also as a potential intermediate for further reaction products. Nevertheless, it can be excluded that the TiC is the only source of carbon for the C_org_ species as the increase in the organic species is significantly higher than the loss in the TiC signal.

**FIGURE 2:**
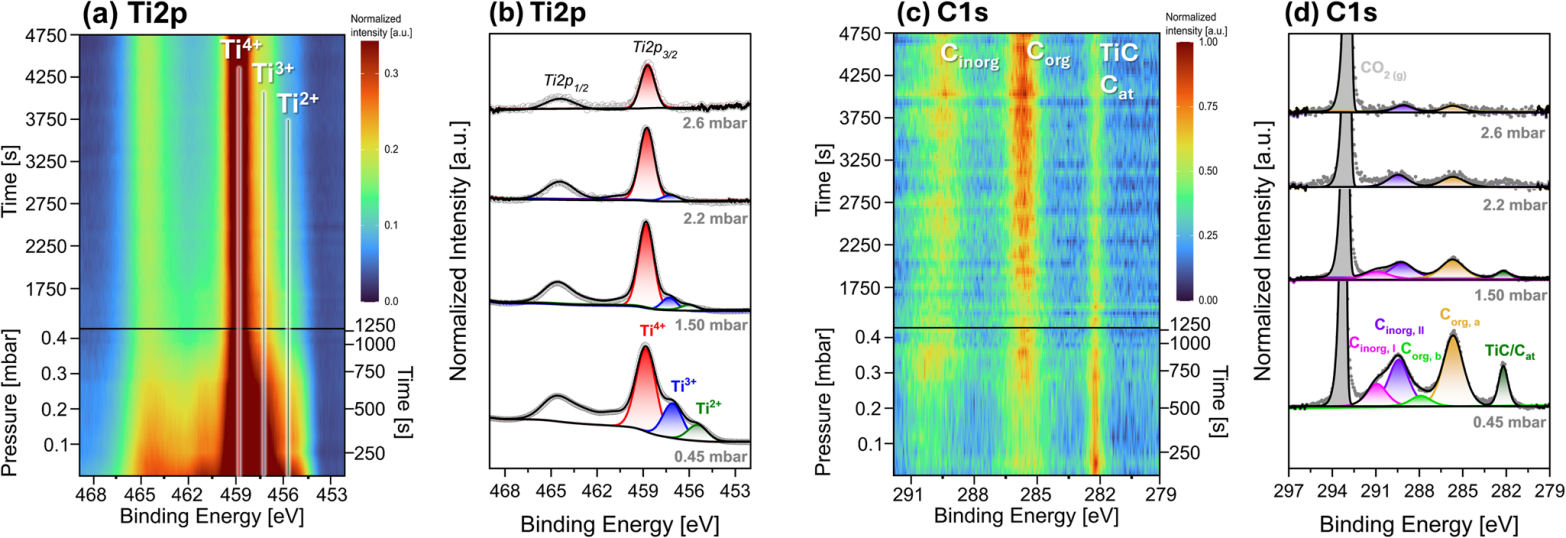
In situ NAP‐XPS core level spectra of highly defective rutile TiO_2_(110) at different pressures of CO_2 (g)_. (a) 3D color plot of the Ti 2p signal at (lower section) increasing pressures up to 0.45 mbar CO_2_ and (upper section) over time at a constant pressure of 0.45 mbar CO_2_ (isobar). The corresponding C1s spectrum is shown without the gas phase signal of CO_2_ for increasing pressures (c, lower section) and at constant pressures (c, upper section). Panels (b) and (d) show averaged spectra at different constant pressures for Ti 2p (b) and C 1s (d), respectively.

Surface oxidation by CO_2_ is already strong at low pressures. Figure 2a presents the Ti2p core level spectra as a function of CO_2_ pressures and time, which have been recorded in situ upon continuously increasing the CO_2_ pressure from UHV to 0.45 mbar over 1250 s. Already pressures of 0.1 mbar CO_2_ lead to a reoxidation of the Ti^2+^ species (455.3 eV [green line]) visible as a color change in the plot from dark red to orange. The same applies to the oxidation of the Ti^3+^ species, which cannot be seen directly from Figure 2a due to the color scale used in this plot, chosen to be sensitive for the following isobaric segment. The identical spectrum displayed with a different color scale can be found in Figure S1 in the supporting information. At the final pressure of 0.45 mbar Ti^4+^ is the dominant oxidation state. After the pressure ramp, we added an isobaric segment and monitored the ongoing slower oxidation of the remaining Ti^3+^ and Ti^2+^, which is apparent from the slow intensity loss for the two reduced Ti signals from 1250 to 4750 s (from green to blue for Ti^2+^ and from orange to yellow/green for Ti^3+^).

As shown in Figure 2b, we exposed the sample to higher pressures of CO_2_ (same sample after the isobaric experiment at 0.45 mbar). In the Ti2p core level spectra at 0.45 mbar, the expected signals of all three already discussed Ti^n+^ species are observed. At higher pressures of 1.50 mbar, the defect states (Ti^3+^ and Ti^2+^) are significantly diminished (70% less for Ti^2+^, 44% less for Ti^3+^ relative to Ti^4+^) compared to 0.45 mbar but can still be deconvoluted. When the pressure is further increased to 2.2 mbar, only a small Ti^3+^ shoulder remains visible. The last pressure increase to 2.6 mbar finally shows the loss of a significant Ti^3+^ peak, leaving only a Ti^4+^ signal. Due to the increased electron scattering and signal attenuation with increasing pressure, the signal‐to‐noise ratio is increasingly deteriorated. Nevertheless, the obtained signal‐to‐noise ratio is low enough to allow reliable identification of Ti^3+^ or Ti^2+^ densities exceeding 10%. The observed significant loss of Ti^3+^ and Ti^2+^ is thus not due to reaching a detection limit. At the same time, the increased coverage of the surface with carbon should be considered when discussing changes in intensity, as the relative C coverage increased by a factor of ∼4 from 0.45 to 2.6 mbar.

Interestingly, a post‐mortem experiment reveals the partial reversibility, as a small amount of Ti^3+^ is detected on the sample after evacuation of the gas. Comparison of the Ti2p core level spectra in 2.6 mbar (Figure 2b, top spectrum) and after pumping out the CO_2_ (Figure 1a, bottom spectrum) indicates a pressure‐dependent reversible reactive adsorption of the CO_2_ on TiO_2_. This behavior matches the general assumption of defects in titania existing as mobile small polarons that can be easily quenched in the near‐surface region, as recently shown in a combined XPS and NEXAFS study of phenylphosphonic acid by Lytken and coworkers [43]. As our experiments are performed with high surface sensitivity, adsorbate‐induced migration of polarons from the surface to the subsurface and vice versa could be a possible reason for the pressure‐dependent (reversible) appearance of Ti^3+^ states in XPS.

The observed reoxidation in a few mbar of CO_2_ at room temperature is comparable to the surface reoxidation by vacuum annealing to temperatures between 673 to 723 K, as we and others have reported for rutile TiO_2_(110) single crystals [28, 44]. However, in contrast to vacuum annealing, we expect that no surface reordering occurs at room temperature. Further, we exclude a relevant loss of defect sites by bulk diffusion because UHV‐level studies regarding the self‐diffusion of defects in rutile TiO_2_ revealed that defects created by annealing are becoming mobile only above 400 K. Even though there is already a significant diffusion of oxygen vacancies between 340 K and 420 K as shown by Zhang et al. using isothermal STM [45], further studies of O adatom formation show that not all oxygen vacancies are healed at 300 K. Du et al. attributed this to a reduction of the reactivity of oxygen vacancies from supplying charges to the O adatoms [46]. Elevated temperatures lead to reoxidation of most defects [47], which has likewise been observed on our samples heated up to 700 K in 1.5 mbar of CO_2_ as shown in Figure S2.

The amount of adsorbed C‐species increases over the duration of the experiment. Figure 2c shows the development of the three carbon species during the CO_2_ pressure ramp and beyond. The C_inorg_ species at ∼289.6 eV slowly builds up while the pressure increases, with the intensity plateauing at pressures of 0.30–0.40 mbar. The same can be observed for the C_org_ species at ∼285.9 eV, which reaches a stable intensity at 0.35 mbar. However, some further intensity increase appears over the duration of the experiment. The carbide species at 282.2 eV is the only signal losing intensity over time, which is widely consumed around 0.35 mbar. In addition, slight intensity oscillations appear as a consequence of ring current oscillations due to top‐up injections from the linear accelerator.

Higher CO_2_ pressures up to 2.6 mbar yield analogous results in the C1s region compared to smaller pressures (∼0.1–0.45 mbar). Figure 2d displays the C1s spectra for increasing pressures. At 0.45 mbar, the presence of similar species as in Figure 1b (which was a post‐mortem measurement) can be confirmed in situ: the spectrum is dominated by the gas phase signal at 293.1 eV. At ∼290 eV, there is a signal that we attribute to C_inorg_. However, in contrast to Figure 1b, we are now able to deconvolute the signal into two species due to the higher concentration of these species under in situ conditions: a C_inorg,I_ (magenta, denotes CO_3_^2−^, CO, and formate [HCO_2_^−^]) species at 291.0 eV and a C_inorg,II_ (purple, HCO_3_^−^) at 289.5 eV. An additional compound at higher binding energies might contribute to the carbonate signal, like a CO_2_^δ−^ species that was observed on Ag/TiO_2_ nanoparticles with NAP‐XPS [38]. Next to the (bi)carbonate, the already introduced organic species (C_org a_ and C_org b_) are present at 285.7 eV and 287.9 eV but cannot be further resolved. At 282.1 eV, the carbide species is present. While increasing the pressure to 1.5 mbar, the total intensity of all species is reduced due to increased electron scattering, with C_org,b_ not being distinguishable anymore due to the high noise.

Summarizing these results, it can be stated that CO_2_ is activated via oxidizing defective TiO_2_ at low mbar pressures. Hereby, the oxidation seems to be partially reversible and shows a pressure dependence, as some of the defects oxidized at 2.6 mbars reappear in the post‐mortem experiments in UHV.

### 2.2 Population of Reaction Pathways in the Presence of H‐Donors: Water as a Reaction Mediator

In this section, we focus on the role of hydrogen donors for the formation of surface intermediates from activated CO_2_, which span up a chemical network with different (interconnected) reaction pathways. The presence of key intermediates can be seen as a fingerprint for the population of different reaction pathways, that is, the promotion or suppression of selected reaction steps in various gas environments.

**FIGURE 3:**
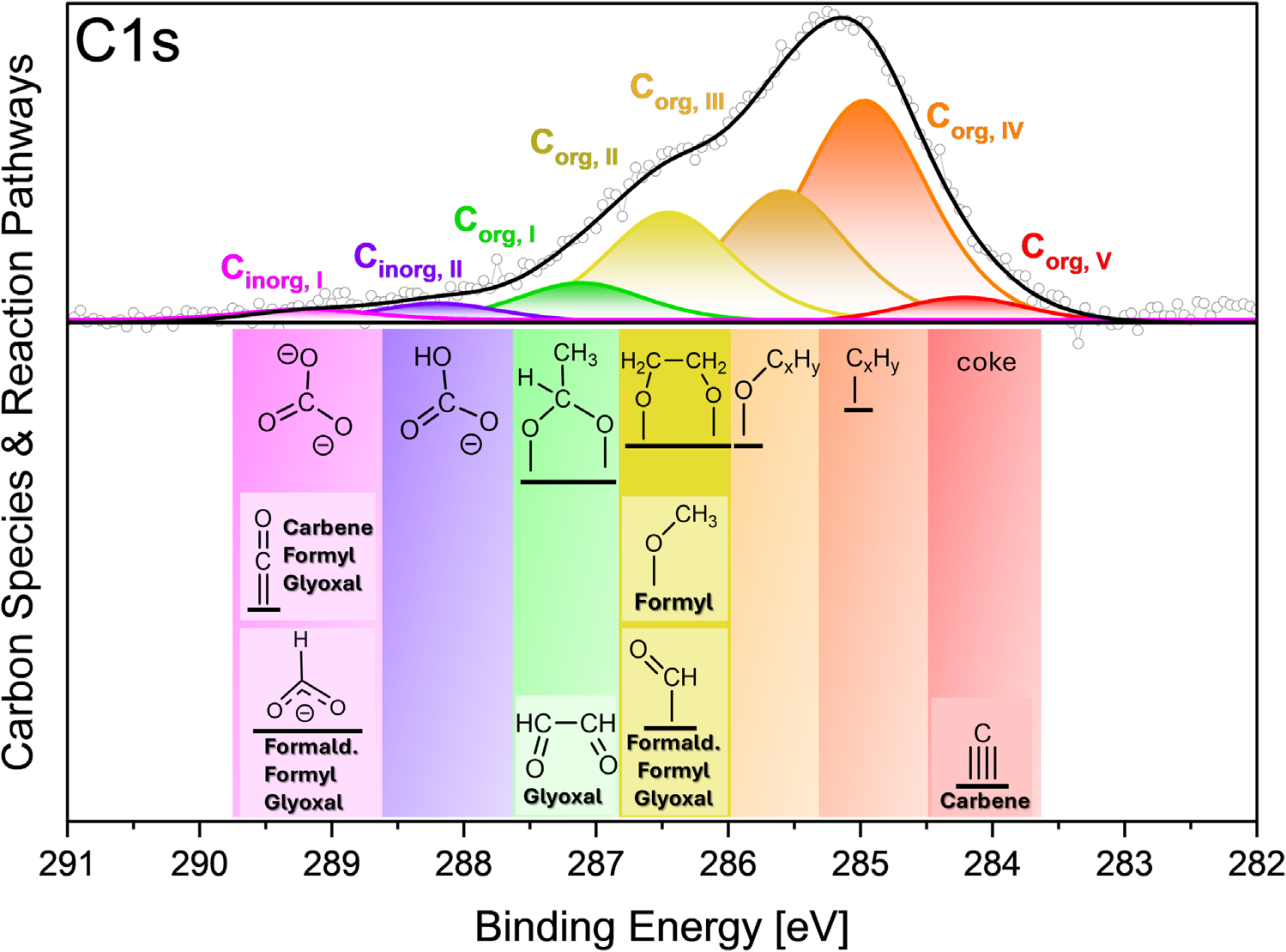
Exemplary NAP‐XPS C1s spectrum of surface intermediates present during CO_2_ activation in 1.5 mbar CO_2_ + 1.5 mbar H_2_O at 450 K. The assignment of pathways was performed according to reference [48]. References for further species assignment can be found in Table 1. Characteristic intermediates from the four relevant CO_2_ activation pathways, which therefore are a fingerprint of the population of different reaction pathways, are displayed in a brighter box.

In different gas mixtures, the composition of surface intermediates varies. Due to a better signal‐to‐noise ratio and distinct shoulders forming in the spectral signature during a temperature ramp, we are now able to assign species to different surface intermediates. The distribution of these species can be considered as a descriptor for the population of different CO_2_ reaction pathways for reduction to C_1_ and C_2_ products as described in the literature [48]. In Figure 3, an example C1s spectrum is displayed with a literature‐based assignment of surface species and key intermediates from the four commonly relevant pathways for CO_2_ reduction (in brighter boxes), which have to be considered herein. These pathways are (1) the formaldehyde‐pathway, (2) the carbene‐pathway, (3) the formyl‐pathway, and (4) the glyoxal‐pathway as discussed in multiple studies in literature [49, 50] and summarized by Y. Wang et al. [48]. Indeed, the spectra indicate the parallel population of multiple reaction pathways, which is not surprising considering the heterogeneous nature of the sample, containing diverse reaction sites (Ti^n+^, O_b_, O_b,vac_) and the presence of subsequent reaction channels with water.

Seven different carbon species on the surface can be distinguished in the C1s spectra, with changing quantities responding to the experimental conditions. The additional gas phase signal at 293.0 eV is not shown to be more sensitive to the smaller surface intermediate signals. The signal at 289.6 eV (magenta), labeled C_inorg,I_, is assigned to carbonates, carbon monoxide, or possibly formate as a central intermediate in all the reaction pathways. The presence of formate in an H‐deficient reaction environment could be caused by the presence of background water contaminations leading to surface hydroxyls. This cannot be completely excluded due to the nature of the used high‐pressure setup. The signal C_inorg,II_ at 288.6 eV (purple) denotes bicarbonate as discussed further above. For the wide signal complex between 284 eV and 288 eV, five different species were identified. Due to the finite chemical resolution of XPS and the complexity of multiple possible intermediates, we cannot directly assign one certain compound or bond type for each species. It has to be considered that XPS as a stand‐alone method cannot further distinguish between the specific species grouped together in the same deconvolution component (color‐coded). However, based on their position, groups of chemical functionalities can be assigned. Table 1 gives an overview of the observed signals herein and their common assignment based on the literature.

**TABLE 1:** Peak assignment for overserved carbon species and their C1s binding energies.

Species	C1s binding energy	Peak assignment/references
CO_2_ (g)	293.0 eV	CO_2_ gas phase [25, 26]
C_inorg,I_	289.6 eV	Carbonate [38], carbon monoxide [40], formate [51]
C_inorg,II_	288.6 eV	Bicarbonate [38, 39]
C_org,I_	287.2 eV	Dioxyethylene (from EtOH) [41]
C_org,II_	286.4 eV	Diolate [52], alkoxy (O‐bound carbon) [53], methoxy [54, 55]
C_org,III_	285.6 eV	Aliphatic rests of oxygenates [53]
C_org,IV_	284.9 eV	C‐C/C‐H/adventitious carbon [56]
C_org,V_	284.2 eV	Coke [57], carbenes

A species C_org,I_ (green), peaking at 287.2 eV, is attributed to carbon‐containing molecules rich in oxygen, where the signal corresponds to carbon atoms directly bonded to oxygen. A similar assignment can be found, for example, for NAP‐XPS studies of ethanol on TiO_2_, where a dioxyethylene species was attributed to a signal at 287.76 eV [41]. This type of molecule can also be considered a precursor in the glyoxal pathway, leading to a variety of C‐C‐coupling products. The species C_org,II_ (light yellow) at 286.4 eV is also attributed to organic oxygenates, but with less oxygen directly bound to that carbon. A possible ethylene species formed from diolate intermediates was assigned to 286.6 eV in studies on UHV‐level photo‐ and thermocatalysis of formaldehyde on rutile TiO_2_(110) [52]. Similar assignments can be made for intermediates in the formyl and formaldehyde pathway, like methoxy [54, 55] (but also theoretically other alkoxy groups [53]) and formyl groups [52, 58].

The third species C_org,III_ in a darker yellow color with a binding energy of 285.6 eV can be assigned to organic carbon in sp^3^ hybridization in the aliphatic rests of oxygenates such as alcohols. For example, the terminal methyl group in ethanol has been observed in NAP‐XPS at 285.3 eV [41]. Similar assignments have been made in UHV‐XPS for several aliphatic alcohols like ethanol (285.2 eV), isopropanol (285.2 eV), or n‐propanol (∼285 eV; the exact C‐atoms could not be distinguished) [53]. However, it can be excluded that the signal is an α‐CH_x_ directly bound to oxygen, as these alkoxy‐type species show higher binding energies of 286.6 eV (ethanol), 286.5 eV (propanol), or 286.9 eV (methanol) [53]. The fourth organic species in orange, C_org,IV_ is located at 284.9 eV and can be assigned to C‐C/C‐H species similar to adventitious carbon, as discussed in a multi‐user facility review by M.C. Biesinger [56]. Finally, the last species in red at 284.2 eV (C_org,V_) denotes coke/graphitic carbon on the surface. This signal would be a main indicator for the population of the carbene pathway, accompanied by a CO signal lying under the C_inorg,I_ signal.

**FIGURE 4:**
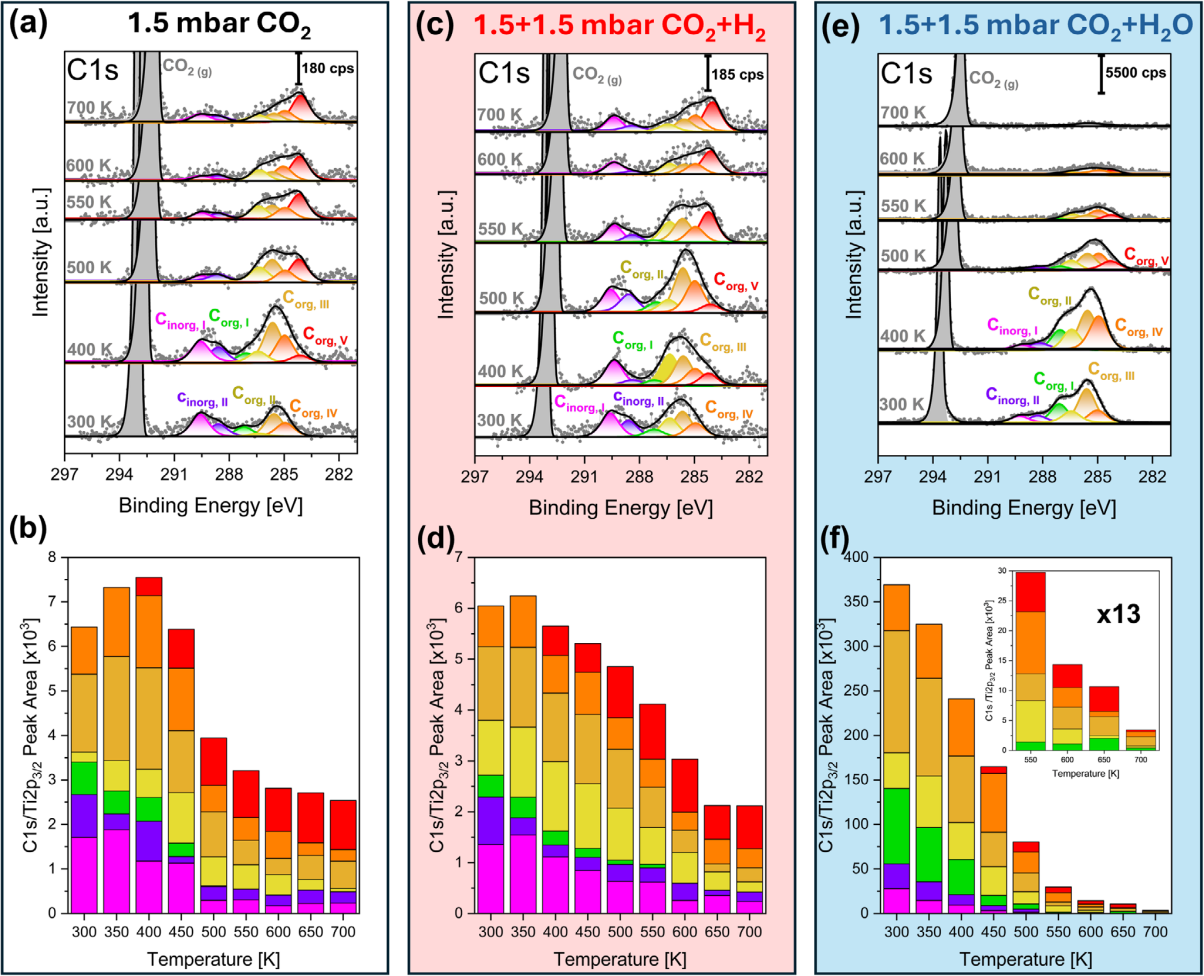
In situ NAP‐XPS C1s spectra of highly defective rutile TiO_2_(110) in different gas mixtures ([a, b] 1.5 mbar CO_2_, [c, d] 1.5 mbar CO_2_ + 1.5 mbar H_2_ and [e, f] 1.5 mbar CO_2_ + 1.5 mbar H_2_O) at increasing temperatures, and the quantitative development of C‐species relative to the Ti2p_3/2_ integral (bar graphs) to compensate for intensity changes due to e.g. thermal expansion of the sample. All spectra are background‐subtracted and shifted in height.

All mentioned signals are present in all three gas combinations of CO_2_, CO_2_ + H_2_ and CO_2_ + H_2_O, but vary in intensity and their temperature‐dependence. The quantitative development of C1s species is shown in Figure 4. Some of the shown spectra exhibit a strong total increase of some species, for example, C_org,IV_, that are not as evident from the bar charts. Thermal expansion of the sample in front of the analyzer cone, inconstant ring current, or variation in general sample position can cause significant changes in the overall intensity (which affects the overall intensity of the shown spectra). Therefore, the quantitative evaluation shown by the bar charts includes normalization with respect to the Ti2p_3/2_ signal and reflects the actual coverage by C‐containing species. Some temperatures in the bar graph are not shown as spectra in Figure 4; these can be found in Figure S3. Due to their similarity, we will discuss the samples in pure CO_2_ and in a CO_2_ + H_2_ mixture (1:1, 1.5 mbar each) together. In pure CO_2_ (white box) and CO_2_ + H_2_ (red box), shown in Figure 4a–d, C_inorg_ and C_org,III_ and C_org,IV_ are the dominating species. While heating, a C_org, V_ component is formed at 400 K, and a maximum of carbon coverage is reached at 350–400 K. The overall amount of carbon decreases visibly when the sample is further heated up to 700 K. Most significant changes can be observed at 400‐600 K, at temperatures correlated to defect mobility [47]. From the available data, it remains unclear whether this is due to desorption of CO_2_ or product formation. Both experiments exhibit similar surface coverage, implying that hydrogen has no relevant effect on product formation.

After H_2_O was added to the CO_2_ gas environment (Figure 4e,f), a significant increase in the amount of adsorbed C‐species was observed, approximately by a factor of 50 compared to pure CO_2_ or mixtures of CO_2_ and H_2_ at room temperature. The relative contribution of C_inorg_ is reduced, even though the absolute amount is still increased by an order of magnitude compared to pure CO_2_. When water is present, two temperature regimes can be distinguished.

**FIGURE 5:**
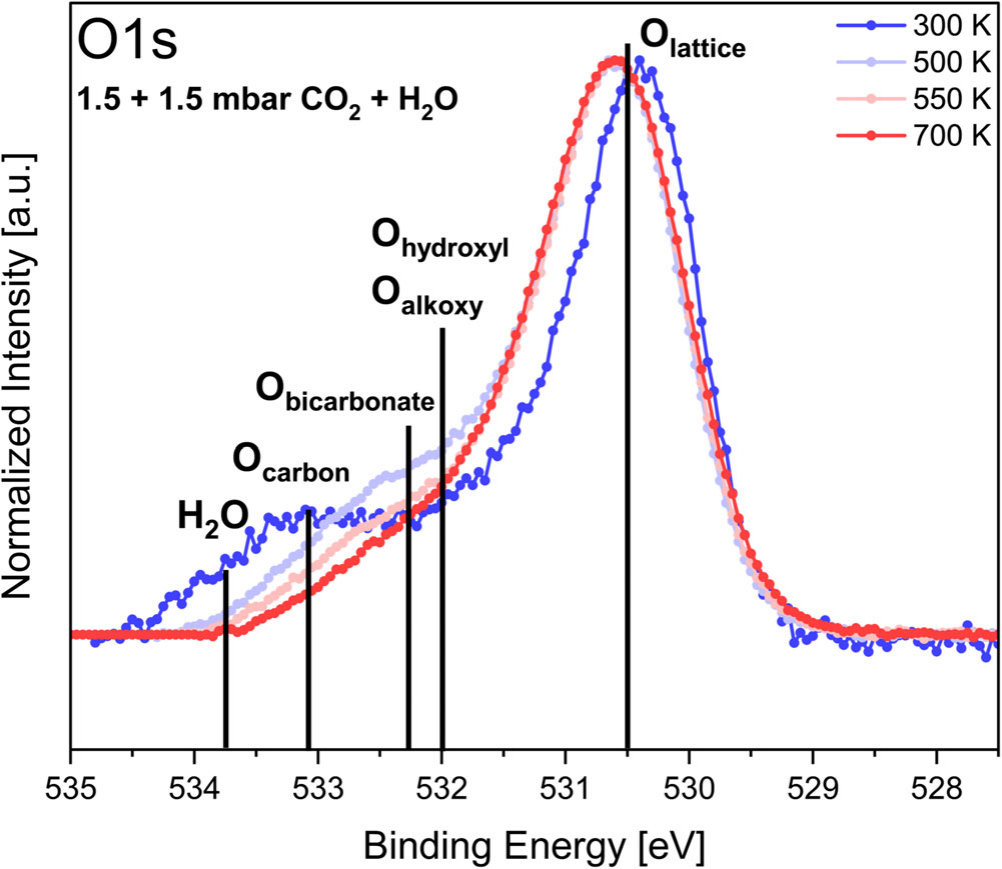
O1s spectrum of the CO_2_ + H_2_O sample at different temperatures. The spectra are background subtracted and normalized.

The first regime at lower temperatures of 300–500 K is characterized by the predominantly increased species C_org,I_ and C_org,III_ along with the copresence of surface hydroxyls. This indicates that oxygen‐rich organic compounds directly derived from CO_2_ are present and oxygen‐rich pathways are promoted. The corresponding O1s spectrum (Figure 5) supports this finding. However, the oxygen species present in intact organic compounds (O_carbon_, ∼533 eV) like ethanol [41] diminish upon heating to temperatures of 500 K, showing the same behavior as their corresponding C1s signals (C_org,I_ and C_org,III_).

A parallel loss of hydroxyl species (O_hydroxyl_, ∼532 eV) was observed around 500–550 K. In an XPS study investigating water adsorption on different TiO_2_ nanoparticles, powders, and single crystals, such OH signals were observed peaking at 532.2 eV. In NAP‐XPS studies of methanol [55] and ethanol [41] on TiO_2,_ OH groups peaking at ∼531.7–532.0 eV were reported to overlap with the O of alkoxy species resulting from deprotonation of the alcohols. These surface hydroxyls act as efficient H and OH donors, promoting the three oxygen‐rich pathways. As these three pathways show similar spectral signatures and share common surface intermediates (e.g., the formyl monomer in the formaldehyde and glyoxal pathway), we cannot distinguish between these three pathways. Only the carbene pathway can be excluded as a dominating process in the low‐temperature regime, which would require a number of oxygen removal steps.

Within this first temperature interval, heating to 400 K leads to a decrease of all C‐species, mainly in the C_inorg_ features and the initially more present C_org,I_ and C_org,III_ species. This also corresponds to the loss of the O1s shoulder at ∼532.3 eV that could originate from bicarbonate/hydroxy‐carbonate being formed by the reaction of OH and CO_2_. The signal position has been assigned in the past by Mirabella and coworkers in their study of CO_2_‐acidified water on Fe_3_O_4_ and TiO_2_ [39]. Thus, the presence of water significantly increases the surface coverage of activated intermediates from CO_2_, potentially as a mediator through surface hydroxyls to form inorganic carbon species as precursor‐like adsorbates on the surface. This corresponds to TPD studies in UHV [15], showing that adsorption of a CO_2_ + H_2_O mixture on TiO_2_ leads to formation of bicarbonate, while this reaction is suppressed for stepwise adsorption of CO_2_ followed by water, which leads to displacement of CO_2_ by water.

The temperature interval around 500–550 K appears as a chemical threshold. While lower temperatures favor oxygen‐rich intermediates and pathways on the hydroxylated surface, the loss of hydroxyls apparent from the O1s spectra above 500 K triggers the formation of carbon/carbene intermediates (C_org,V_), along with a significant overall loss of carbon adsorbates, being more than halved for all species. With the removal of hydroxyls as potent H sources, the chemistry above 550 K becomes similar to the experiments in pure CO_2_ and CO_2_/H_2_. Above 550 K, the gradual loss of carbon continues, finally reaching similar intensity levels as for the experiments in pure CO_2_ and CO_2_/H_2_, indicating a convergence of surface reactions. At this point in the absence of OH, the carbene pathway becomes dominant, leading to coke formation on the surface due to lack of reactive hydrogen. Table 2 gives an overview of the O1s regions and their assigned species, including references.

**TABLE 2:** Peak assignment for observed oxygen species and their O1s binding energies.

Species	O1s binding energy	Assignment/references
O_lattice_	530.5 eV	Bulk TiO_2_[44]
O_hydroxyl_/O_alkyl_	531.7–532.2 eV	Hydroxyls [59] and deprotonated alcohols [41, 55]
O_bicarbonate_	532.3 eV	Bicarbonates/hydroxycarbonates [60]
O_carbon_	533.2 eV	Intact organic molecules, for example, alcohols [41, 55]
H_2_O	533.7 eV	Water, not dissociated [59]

**FIGURE 6:**
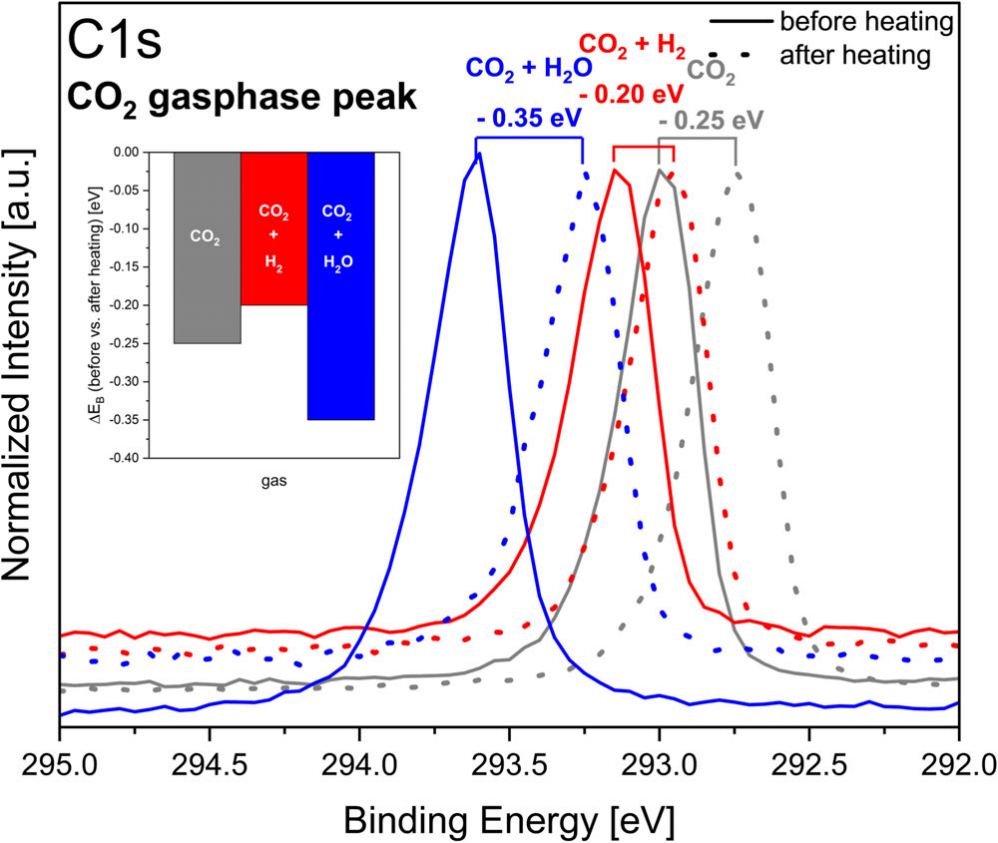
C1s spectra of the CO_2_ gas phase peak in three different gas mixtures, showing the shift of the gas phase signal at 300 K (straight line) versus 700 K (dashed line) due to sample work function changes. The insets show the change of work function as a bar chart.

The role of surface hydroxyls is manifested in work function changes. As the sample and the detector are grounded with each other, shifts in gas phase signals can be utilized to monitor changes of the sample work function [61]. An apparent positive shift in binding energy of gas phase signals denotes a decrease of the sample work function. Herein, the CO_2_ gas phase signal is used to discuss absolute changes of the work function. Figure 6 shows the gas phase CO_2_ at 300 K (bold lines) for all three gas mixtures on individual positions. The sample in pure CO_2_ has the lowest peak position (∼293.0 eV) and therefore the highest work function. Addition of H_2_ lowers the work function only very slightly (∼−0.1 eV, positive shift of the gas phase signal), while adding water to the CO_2_ atmosphere lowers the work function significantly (∼−0.6 eV compared to pure CO_2_). Upon heating to 700 K, all signals are shifted to lower binding energies (dashed lines) by 0.9–1.2 eV, indicating higher work functions in situ. Here the relative change within CO_2_/water is the strongest, reaching similar final levels like in CO_2_/H_2_. This supports the convergence of the surface chemistry at elevated temperatures in all atmospheres due to the desorption of hydroxyls above 550 K.

Surely, there is a chemical and a thermal contribution in these in situ measurements. The surface termination of titania has a significant impact on the system's work function. In general, the work function is decreased when electrons are accumulated on the surface either by reduction, for example, through sputtering, or by adsorption of electron‐donating molecules like water, as shown by ultraviolet photoelectron spectroscopy (UPS) and metastable electron impact spectroscopy (MEIS) studies on rutile titania [61]. Table 3 shows examples of the respective surface termination and the corresponding work function of rutile TiO_2_(110). The presence of surface hydroxyls decreases the work function by 0.3 eV compared to slightly reduced samples, meaning that an increase in binding energy of the CO_2_ gas phase signal should be observed. In our case, however, we observe the opposite behavior. This is explainable with the strongly oxygen‐deficient nature of our sample (dominant Ti^3+^ and Ti^2+^). This higher degree of reduction leads to a significantly lower initial work function of the sample compared to a slightly reduced sample, thus showing an increase in work function when being hydroxylated.

**TABLE 3:** Overview of TiO_2_ surface terminations and corresponding work functions (WF).

Termination	Description	WF [eV]	Ref.
h‐TiO_2_	Vacancies filled with OH	4.9	[61]
r‐TiO_2_	6%–9% oxygen vacancies	5.2	[61]
o‐TiO_2_	Oxygen adatoms on a stoichiometric surface	5.35	[61]
qs‐TiO_2_	Stoichiometric surface with close to no defects	5.5	[61]

The work function increase upon heating can be explained by two factors. The first is the thermal (and chemical) reoxidation due to the defect mobility in TiO_2_ at elevated temperatures [47]. However, the change in work function is stronger for the CO_2_/H_2_O sample than the CO_2_ sample, and the work functions at 700 K have a smaller difference. Therefore, the loss of hydroxyls at higher temperatures is not only dominating the population of CO_2_ reduction pathways but also dominates the work function in situ, with consequences, for example, for photo‐ or electrochemical applications.

## 3 Conclusion

To summarize, we followed the activation of CO_2_ on the highly defective TiO_2_(110) surface in situ over different thermal and pressure regimes, showing that only small pressures are needed to reoxidize most surface defects. At the same time, defects located in the subsurface remain more stable at room temperature in a few mbars of CO_2_ but react at higher pressures (> 1 mbar). In the absence of efficient H and OH donors (pure CO_2_, or CO_2_ + H_2_ without potent H_2_ dissociation sites), all four common reaction pathways appear parallelly populated, but coking (potentially along the carbene pathway) becomes the dominant process for higher temperatures.

The presence of surface hydroxyls from water drastically changes the surface chemistry and boosts the amount of activated carbon intermediates at the surface, favoring oxygen‐rich pathways. Therefore, the formation of inorganic carbon species and oxygen‐rich organic molecules is significantly promoted by an aqueous atmosphere. Under these conditions, the carbene pathway is suppressed and plays only a minor role. The loss of surface hydroxyls above 550 K triggers the onset of coke formation, along with a severe loss of carbon intermediate coverage and a significant increase in work function. For *T* > 550 K, the surface chemistry converges to the case of water‐free conditions (CO_2_, CO_2_ + H_2_), with coking (along the carbene pathway) becoming dominant at elevated temperatures.

## Author Contributions

**Justin Klimek**: investigation (lead), formal analysis (lead), visualization (lead), writing – original draft (lead). **Filip Hallböök**: investigation (supporting). **Niko Kruse**: investigation (supporting). **Fangliang Li**: investigation (supporting). **Sara Blomberg**: funding acquisition. **Katharina Al‐Shamery**: conceptualization, supervision, funding acquisition, writing – review and editing. **Lars Mohrhusen**: conceptualization (lead), project administration (lead), supervision (lead), funding acquisition (lead), writing – review and editing.

## Conflicts of Interest

The authors declare no conflicts of interest.

## Supporting information




**Supporting File 1**: The following files are available free of charge: The experimental section and Ti2p core level spectra showing a different color gradient in the 3D plot and Ti2p core level spectra (2D) comparing temperatures of 300 and 700 K, C1s core level spectra of the gas mixtures at 350, 450, and 650 K can be found in the supporting information (PDF).

## Data Availability

The experimental data used within this work is available from the authors upon reasonable request.

## References

[1] Z. Wu , A. Vermeulen , Y. Sawa , et al., “Investigating the Differences in Calculating Global Mean Surface CO_2_ Abundance: The Impact of Analysis Methodologies and Site Selection,” Atmospheric Chemistry and Physics 24 (2024): 1249–1264, 10.5194/acp-24-1249-2024.

[2] S. Chu and A. Majumdar , “Opportunities and Challenges for a Sustainable Energy Future,” Nature 488 (2012): 294–303, 10.1038/nature11475. 22895334

[3] T. P. Senftle and E. A. Carter , “The Holy Grail: Chemistry Enabling an Economically Viable CO_2_ Capture, Utilization, and Storage Strategy,” Accounts of Chemical Research 50 (2017): 472–475, 10.1021/acs.accounts.6b00479. 28945424

[4] R. Guil‐López , N. Mota , J. Llorente , et al., “Methanol Synthesis From CO_2_: A Review of the Latest Developments in Heterogeneous Catalysis,” Materials 12 (2019): 3902, 10.3390/ma12233902. 31779127 PMC6926878

[5] Z. Zhang , Z. Yang , L. Liu , Y. Wang , and S. Kawi , “Catalytic CO_2_ Conversion to C_1_ Chemicals Over Single‐Atom Catalysts,” Advanced Energy Materials 13 (2023): 2301852, 10.1002/aenm.202301852.

[6] V. Dieterich , A. Buttler , A. Hanel , H. Spliethoff , and S. Fendt , “Power‐to‐Liquid via Synthesis of Methanol, DME or Fischer–Tropsch‐Fuels: A Review,” Energy & Environmental Science 13 (2020): 3207–3252, 10.1039/D0EE01187H.

[7] O. Shtyka , R. Ciesielski , A. Kedziora , et al., “Photocatalytic Reduction of CO_2_ over Me (Pt, Pd, Ni, Cu)/TiO_2_ Catalysts,” Topics in Catalysis 63 (2020): 113–120, 10.1007/s11244-020-01241-y.

[8] P. Nuss and M. J. Eckelman , “Life Cycle Assessment of Metals: A Scientific Synthesis,” PLoS ONE 9 (2014): e101298, 10.1371/journal.pone.0101298. 24999810 PMC4085040

[9] J. Strunk , Metal Oxides in Energy Technologies (Elsevier, 2018), 275–301.

[10] S. Tan , Y. Zhao , J. Zhao , et al., “CO_2_ Dissociation Activated Through Electron Attachment on the Reduced Rutile TiO_2_(110)‐1 × 1 Surface,” Physical Review B 84 (2011): 155418, 10.1103/PhysRevB.84.155418.

[11] A. Naldoni , M. Altomare , G. Zoppellaro , et al., “Photocatalysis With Reduced TiO_2_: From Black TiO_2_ to Cocatalyst‐Free Hydrogen Production,” ACS Catalysis 9 (2019): 345–364, 10.1021/acscatal.8b04068. 30701123 PMC6344061

[12] C. P. León , K. Sagisaka , D. Fujita , and L. Han , “Ethanol adsorption on rutile TiO_2_(110),” RSC Advances 4 (2014): 8550–8557, 10.1039/C3RA47369D.

[13] L. Bodek , K. Buda , P. Ciochon , and B. Such , “Adsorption Behavior of 9‐Anthracenecarboxylic Acid on (110) Rutile TiO_2_ ,” Journal of Physical Chemistry C 126 (2022): 13967–13974, 10.1021/acs.jpcc.2c03549.

[14] J. Kräuter , L. Mohrhusen , F. Waidhas , O. Brummel , J. Libuda , and K. Al‐Shamery , “Photoconversion of 2‐Propanol on Rutile Titania: A Combined Liquid‐Phase and Surface Science Study,” Journal of Physical Chemistry C 125 (2021): 3355–3367, 10.1021/acs.jpcc.0c10734.

[15] M. A. Henderson , “Evidence for Bicarbonate Formation on Vacuum Annealed TiO_2_(110) Resulting From a Precursor‐mediated Interaction Between CO_2_ and H_2_O,” Surface Science 400 (1998): 203–219, 10.1016/S0039-6028(97)00863-7.

[16] T. L. Thompson , O. Diwald , and J. T. Yates , “CO_2_ as a Probe for Monitoring the Surface Defects on TiO_2_(110)Temperature‐Programmed Desorption,” Journal of Physical Chemistry B 107 (2003): 11700–11704, 10.1021/jp030430m.

[17] D. P. Acharya , N. Camillone , and P. Sutter , “CO_2_ Adsorption, Diffusion, and Electron‐Induced Chemistry on Rutile TiO_2_(110): A Low‐Temperature Scanning Tunneling Microscopy Study,” Journal of Physical Chemistry C 115 (2011): 12095–12105, 10.1021/jp202476v.

[18] Y. Cao , S. Hu , M. Yu , S. Yan , and M. Xu , “Adsorption and Interaction of CO_2_ on Rutile TiO_2_(110) Surfaces: A Combined UHV‐FTIRS and Theoretical Simulation Study,” Physical Chemistry Chemical Physics 17 (2015): 23994–24000, 10.1039/C5CP04013B. 26313610

[19] M. Xu , Y. Cao , R. Xu , S. Hu , and S. Yan , “UHV‐FTIRS Studies on Molecular Competitive Adsorption: ^12^CO, ^13^CO and CO_2_ on Reduced TiO_2_(110) Surfaces,” Physical Chemistry Chemical Physics 16 (2014): 23711–23715, 10.1039/C4CP03158J. 25272287

[20] R. S. Smith , Z. Li , L. Chen , Z. Dohnálek , and B. D. Kay , “Adsorption, Desorption, and Displacement Kinetics of H_2_O and CO_2_ on TiO_2_(110),” Journal of Physical Chemistry B 118 (2014): 8054–8061, 10.1021/jp501131v. 24645910

[21] M. Osmić , L. Mohrhusen , and K. Al‐Shamery , “Bulk Defect Dependence of Low‐Temperature Partial Oxidation of Methanol and High‐Temperature Hydrocarbon Formation on Rutile TiO_2_(110),” Journal of Physical Chemistry C 123 (2019): 7615–7626, 10.1021/acs.jpcc.8b02953

[22] J. Kräuter , L. Mohrhusen , T. Thiedemann , M. Willms , and K. Al‐Shamery , “Activation of Small Organic Molecules on Ti^2+^‐Rich TiO_2_ Surfaces: Deoxygenation vs. C–C Coupling,” Zeitschrift für Naturforschung A 74 (2019): 697–707, 10.1515/zna-2019-0135.

[23] L. Mohrhusen and K. Al‐Shamery , “Conversion of Alcohols on Stoichiometric and Reduced Rutile TiO_2_(110): Point Defects Meet Bifunctionality in Oxide (Photo‐)Chemistry,” Catalysis Letters 153 (2023): 321–337, 10.1007/s10562-022-04077-1.

[24] P. M. Clawin , C. M. Friend , and K. Al‐Shamery , “Defects in Surface Chemistry—Reductive Coupling of Benzaldehyde on Rutile TiO_2_(110),” Chemistry— A European Journal 20 (2014): 7665–7669, 10.1002/chem.201402102. 24825761

[25] R. C. E. Hamlyn , M. Mahapatra , D. C. Grinter , et al., “Imaging the Ordering of a Weakly Adsorbed Two‐Dimensional Condensate: Ambient‐Pressure Microscopy and Spectroscopy of CO_2_ Molecules on Rutile TiO_2_(110),” Physical Chemistry Chemical Physics 20 (2018): 13122–13126, 10.1039/C8CP01614C. 29737995

[26] Y. J. Kim , H. Choi , D. Kim , et al., “CO_2_ ‐Driven Oxygen Vacancy Diffusion and Healing on Ti_2_(110) at Ambient Pressure,” Angewandte Chemie International Edition 64 (2025): e202420449, 10.1002/anie.202420449. 39754300

[27] S. Ramanavicius and A. Jagminas , “Synthesis, Characterisation, and Applications of TiO and Other Black Titania Nanostructures Species (Review),” Crystals 14 (2024): 647, 10.3390/cryst14070647.

[28] L. Mohrhusen , J. Kräuter , M. Willms , and K. Al‐Shamery , “Argon Embedded by Ion Bombardment: Relevance of Hidden Dopants in Rutile TiO_2_ ,” Journal of Physical Chemistry C 123 (2019): 20434–20442, 10.1021/acs.jpcc.9b05975.

[29] S. Pétigny , H. Mostéfa‐Sba , B. Domenichini , E. Lesniewska , A. Steinbrunn , and S. Bourgeois , “Superficial Defects Induced by Argon and Oxygen Bombardments on (110) TiO_2_ Surfaces,” Surface Science 410 (1998): 250–257, 10.1016/S0039-6028(98)00297-0.

[30] H. Onishi , K. Fukui , and Y. Iwasawa , “Atomic‐Scale Surface Structures of TiO_2_(110) Determined by Scanning Tunneling Microscopy: A New Surface‐Limited Phase of Titanium Oxide,” Bulletin of the Chemical Society of Japan 68 (1995): 2447–2458, 10.1246/bcsj.68.2447.

[31] K. Szajna , M. Kratzer , D. Wrana , et al., “Influence of TiO_2_(110) Surface Roughness on Growth and Stability of Thin Organic Films,” Journal of Chemical Physics 145 (2016): 144703, 10.1063/1.4964370. 27782523

[32] S. Hashimoto and A. Tanaka , “Alteration of Ti 2p XPS Spectrum for Titanium Oxide by Low‐Energy Ar Ion Bombardment,” Surface and Interface Analysis 34 (2002): 262–265, 10.1002/sia.1296.

[33] W. Göpel , J. A. Anderson , D. Frankel et al., “Surface Defects of TiO_2_(110): A Combined XPS, XAES AND ELS Study,” Surface Science 139 (1984): 333–346, 10.1016/0039-6028(84)90054-2.

[34] R. Gouttebaron , D. Cornelissen , R. Snyders , J. P. Dauchot , M. Wautelet , and M. Hecq , “XPS Study of TiOx Thin Films Prepared by D.c. magnetron Sputtering in Ar‐O_2_ Gas Mixtures,” Surface and Interface Analysis 30 (2000): 527–530, 10.1002/1096-9918(200008)30:1<527::AID-SIA834>3.0.CO;2-Z.

[35] J. Luthin , H. Plank , J. Roth , and C. Linsmeier , “Ion Beam‐Induced Carbide Formation at the Titanium–Carbon Interface,” Nuclear Instruments and Methods in Physics Research Section B: Beam Interactions With Materials and Atoms 182 (2001): 218–226, 10.1016/S0168-583X(01)00679-6.

[36] J. A. Rodriguez , P. Liu , J. Dvorak , et al., “The Interaction of Oxygen With TiC(001): Photoemission and First‐Principles Studies,” Journal of Chemical Physics 121 (2004): 465–474, 10.1063/1.1755669. 15260568

[37] I. Nakamura , M. Sasaki , I. Takano , and Y. Sawada , “Mechanical Properties of Carbon‐Doped TIN Films by Ion Beam Irradiation in Ethylene Gas Atmosphere,” Surface & Coatings Technology 196 (2005): 104–107, 10.1016/j.surfcoat.2004.08.113.

[38] L. Collado , A. Reynal , F. Fresno , et al., “Unravelling the Effect of Charge Dynamics at the Plasmonic Metal/Semiconductor Interface for CO_2_ Photoreduction,” Nature Communications 9 (2018): 4986, 10.1038/s41467-018-07397-2. PMC6255847 30478316

[39] F. Mirabella , J. Balajka , J. Pavelec , et al., “Atomic‐Scale Studies of Fe_3_O_4_(001) and TiO_2_(110) Surfaces Following Immersion in CO_2_‐Acidified Water,” ChemPhysChem 21 (2020): 1788–1796, 10.1002/cphc.202000471. 32639106 PMC7522689

[40] M. Wagstaffe , L. Wenthaus , A. Dominguez‐Castro , et al., “Ultrafast Real‐Time Dynamics of CO Oxidation Over an Oxide Photocatalyst,” ACS Catalysis 10 (2020): 13650–13658, 10.1021/acscatal.0c04098.

[41] R. Jones , G. D'Acunto , P. Shayesteh , F. Rehman , and J. Schnadt , “AP‐XPS Study of Ethanol Adsorption on Rutile TiO_2_(110),” Journal of Physical Chemistry C 126 (2022): 16894–16902, 10.1021/acs.jpcc.2c05389.

[42] A. Mianowski , Z. Robak , M. Tomaszewicz , and S. Stelmach , “The Boudouard–Bell Reaction Analysis Under High Pressure Conditions,” Journal of Thermal Analysis and Calorimetry 110 (2012): 93–102, 10.1007/s10973-012-2334-2.

[43] A. Wolfram , M. Muth , J. Köbl , et al., “Phenylphosphonic Acid on Rutile TiO_2_(110): Using Theoretically Predicted O 1s Spectra to Identify the Adsorption Binding Modes,” Journal of Physical Chemistry C 128 (2024): 12735–12753, 10.1021/acs.jpcc.4c03690.

[44] U. Diebold , “The Surface Science of Titanium Dioxide,” Surface Science Reports 48 (2003): 53–229, 10.1016/S0167-5729(02)00100-0.

[45] Z. Zhang , Q. Ge , S.‐C. Li , B. D. Kay , J. M. White , and Z. Dohnálek , “Imaging Intrinsic Diffusion of Bridge‐Bonded Oxygen Vacancies on TiO_2_(110),” Physical Review Letter 99 (2007): 126105, 10.1103/PhysRevLett.99.126105. 17930526

[46] Y. Du , N. A. Deskins , Z. Zhang , Z. Dohnalek , M. Dupuis , and I. Lyubinetsky , “Formation of O Adatom Pairs and Charge Transfer Upon O_2_ Dissociation on Reduced TiO_2_(110),” Physical Chemistry Chemical Physics 12 (2010): 6337, 10.1039/c000250j. 20532418

[47] M. A. Henderson , “A Surface Perspective on Self‐Diffusion in Rutile TiO_2_ ,” Surface Science 419 (1999): 174–187, 10.1016/S0039-6028(98)00778-X.

[48] Y. Wang , E. Chen , and J. Tang , “Insight on Reaction Pathways of Photocatalytic CO_2_ Conversion,” ACS Catalysis 12 (2022): 7300–7316, 10.1021/acscatal.2c01012. 35747201 PMC9207809

[49] I. A. Shkrob , T. W. Marin , H. He , and P. Zapol , “Photoredox Reactions and the Catalytic Cycle for Carbon Dioxide Fixation and Methanogenesis on Metal Oxides,” Journal of Physical Chemistry C 116 (2012): 9450–9460, 10.1021/jp300122v.

[50] J. Fu , K. Jiang , X. Qiu , J. Yu , and M. Liu , “Product Selectivity of Photocatalytic CO_2_ Reduction Reactions,” Materials Today 32 (2020): 222–243, 10.1016/j.mattod.2019.06.009.

[51] F. B. Nunes , N. Comini , J. T. Diulus , et al., “Dynamic Equilibrium at the HCOOH‐Saturated TiO_2_(110)–Water Interface,” Journal of Physical Chemistry Letters 14 (2023): 3132–3138, 10.1021/acs.jpclett.2c03788. 36952665 PMC10084457

[52] Q. Yuan , Z. Wu , Y. Jin , F. Xiong , and W. Huang , “Surface Chemistry of Formaldehyde on Rutile TiO_2_(110) Surface: Photocatalysis vs Thermal‐Catalysis,” Journal of Physical Chemistry C 118 (2014): 20420–20428, 10.1021/jp5061733.

[53] E. Farfan‐Arribas and R. J. Madix , “Role of Defects in the Adsorption of Aliphatic Alcohols on the TiO_2_(110) Surface,” Journal of Physical Chemistry B 106 (2002): 10680–10692, 10.1021/jp020729p.

[54] D. R. Mullins , M. D. Robbins , and J. Zhou , “Adsorption and Reaction of Methanol on Thin‐Film Cerium Oxide,” Surface Science 600 (2006): 1547–1558, 10.1016/j.susc.2006.02.011.

[55] K. P. Reddy , A. Islam , I. Barba‐Nieto , A. Hunt , I. Waluyo , and J. A. Rodriguez , “The Surface Chemistry of Methanol on TiO_2_(110): Effects of Pressure and Temperature on the Stability of C–O and C–H Bonds,” Journal of Physical Chemistry C 129 (2025): 15637–15645, 10.1021/acs.jpcc.5c04763.

[56] M. C. Biesinger , “Accessing the Robustness of Adventitious Carbon for Charge Referencing (Correction) Purposes in XPS Analysis: Insights From a Multi‐User Facility Data Review,” Applied Surface Science 597 (2022): 153681, 10.1016/j.apsusc.2022.153681.

[57] L. Haug , C. Thurner , M. F. Bekheet , et al., “Pivotal Role of Ni/ZrO_2_ Phase Boundaries for Coke‐Resistant Methane Dry Reforming Catalysts,” Catalysts 13 (2023): 804, 10.3390/catal13050804.

[58] W. Idriss , K. Kim , and M. A. Barteau , “Surface‐Dependent Pathways for Formaldehyde Oxidation and Reduction on TiO_2_(001),” Surface Science (1992): 113–127, 10.1016/0039-6028(92)90464-H.

[59] S. Benkoula , O. Sublemontier , M. Patanen , et al., “Water Adsorption on TiO_2_ Surfaces Probed by Soft X‐Ray Spectroscopies: Bulk Materials vs. Isolated Nanoparticles,” Scientific Reports 5 (2015): 15088, 10.1038/srep15088. 26462615 PMC4604456

[60] S. Axnanda , M. Scheele , E. Crumlin , et al., “Direct Work Function Measurement by Gas Phase Photoelectron Spectroscopy and Its Application on PbS Nanoparticles,” Nano Letters 13 (2013): 6176–6182, 10.1021/nl403524a. 24175587

[61] A. Borodin and M. Reichling , “Characterizing TiO_2_(110) Surface States by Their Work Function,” Physical Chemistry Chemical Physics 13 (2011): 15442, 10.1039/c0cp02835e. 21779605

