# Supplementary material for: Role of Water in Low‐Temperature CO2 Reduction at Defect‐Rich TiO2
Source: Angew Chem Int Ed Engl. 2026 May 13;65(28):e7574479. doi: 10.1002/anie.7574479 (PMC13340491; doi:10.1002/anie.7574479)
Supplement: Supplementary file 1 — Supporting File 1: The following files are available free of charge: The experimental section and Ti2p core level spectra showing a different color gradient in the 3D plot and Ti2p core level spectra (2D) comparing temperatures of 300 and 700 K, C1s core level spectra of the gas mixtures at 350, 450, and 650 K can be found in the supporting information (PDF). [file ANIE-65-e7574479-s001.pdf]

Role of Water in Low-Temperature CO_2 Reduction at Defect-Rich TiO_2

Supplementary Information

Justin Klimek [1], Filip Hallböök [2], Niko Kruse [1], Fangliang Li [1], Sara Blomberg [2], Katharina Al-Shamery [1], Lars Mohrhusen [1]*

[1] Institute of Chemistry, Carl von Ossietzky Universität Oldenburg, Carl-von-Ossietzky-Straße 9-11, 26129 Oldenburg, Germany

[2] Department of Process and Life Science Engineering, Lund University, Naturvetarvägen 24, 22362 Lund, Sweden

METHODS

Materials & Sample Preparation

Rutile TiO_2 (110) single crystals (1 x 10 x ~ 5 mm) were obtained from Surface Net GmbH and mounted onto a stainless-steel flag-style sample holder using stainless steel wire strips. Gold foil was placed underneath the single crystal to increase thermal conductivity. A K-type thermocouple was spot-welded to the sample holder close to the single crystal. All samples were initially degassed in the preparation chamber (base pressure < 5 x10^{-9} mbar) to temperatures up to 850 K. The samples then were cleaned by multiple cycles of Ar^+ sputtering (~300 – 400 K, 10^{-5} mbar, 2000 eV, ~3-5 μA/cm^2, for 20 minutes) and annealing (UHV, 800 K for 30 minutes). For the highly reduced samples used herein, the preparation sequence ended with a sputtering step. All samples were transferred in UHV to the (NAP)-XPS measurement position. The sample quality and cleanliness were confirmed by UHV XPS spectra before further use.

Near-ambient pressure X-ray photoelectron spectroscopy (NAP-XPS)

All data were collected at the solid-gas branch of the HIPPIE beamline at the MAX IV laboratory in Lund, Sweden[1]. The beamline is equipped with a Scienta Omicron HiPP3 electron analyzer and a 2D CCD type detector. Sample heating was performed with an IR Laser through a hole in the sample holder, shining onto a thin stainless-steel foil as heat absorber, on which the gold foil and the TiO_2 sample were placed. This allowed to record *in-situ* spectra in all given atmospheres at temperatures from 300 K to 700 K. Liquid nitrogen cooling traps were used along with dedicated contaminant gas traps to avoid gas contaminations. The purity and composition of the gas mixture was continuously monitored with mass spectrometry at the NAP cell inlet, outlet and first pumping stage of the analyzer, respectively. No relevant traces of

organic contaminants were detected in the gas feeds. Regular checks for beam damage were performed by moving the surface along the focus spot of the analyzer.

Two types of experiments were performed in this work:

1.) A highly defective Rutile TiO_2 (110) single crystal was exposed to different pressures of CO_2 at room temperature (Figures 1 and 2). For this type of experiment, we continuously recorded C1s and Ti2p spectra (photon energy of 600 eV and a slit size of 30 μm). Collection of a C1s spectrum took 57 s, while Ti2p spectra were recorded in 29 s, leading to an average loop duration of 86 s, starting in UHV (13 minutes / 7 sets of spectra), before the CO_2 pressure was slowly increased to 0.45 mbar in a ramp of 20 minutes. The sample was then left in 0.45 mbar of CO_2 atmosphere for 1 hour. After that, the pressure was increased to 1.5 mbar over 30 min. At this pressure another isobaric section of 30 minutes followed before the pressure was increased to 2.6 mbar over another 30 minutes. In the next step, the near-ambient pressure-cell (NAP-cell) was slowly evacuated over a 50-minute period, leading to a final pressure of ~1 x 10^{-5} mbar in the NAP-cell. The local pressure at the sample was monitored by the QMS signal from the first pumping stage of the analyzer, precalibrated from steady-state measurements using a baratron gauge.

2.) Temperature-dependent *in-situ* measurements on the Rutile TiO_2 (110) samples in three different gas environments: 1.5 mbar CO_2, 1.5 mbar CO_2 + 1.5 mbar H_2 and 1.5 mbar CO_2 + 1.5 mbar H_2O (figures 3–6). A freshly prepared (sputtered) sample was used for each experiment. After collection of UHV spectra, the NAP-cell was filled with the gas mixture and a set of core level spectra was collected at 300 K. Then the samples were heated to T = 700 K in steps of 50 K, with core level spectra being collected after every heating step. For these experiments surface-sensitive O1s spectra were collected at 670 eV photon energy, Ti2p at 600 eV, C1s spectra at 370 eV (all with 20 μm slit size) and valence band spectra at 260 eV photon energy (40 μm slit size). More bulk sensitive O1s and Ti2p spectra were collected at

1600 eV photon energy. In addition, O2s core level spectra at each photon energy were collected and used for energy calibration.

Fit and data treatment

All data collected was converted into a VAMAS file (.vms) and processed with CasaXPS Version 2.3.26PR1.0 (Casa Software Ltd). All shown conventional core level spectra were fitted with either a linear (C1s, O1s) or a Shirley background (Ti2p). Components were fitted with Gaussian-Lorentzian products with different Gaussian-Lorentzian ratios, depending on the core level spectrum (mostly GL(30) O1s, GL(5) for Ti2p and GL(50) for C1s). Data were then transferred to Origin for finalizing the graphics. The spectra are either shown including the applied background or after background subtraction, as indicated in the figure caption. Normalization of spectra was performed by division through the intensity of the peak maximum.

For 3D spectra (figure 2) unmodified data were processed using a R Studio script. To allow better distinction between the different titanium species, the color scale was set to an interval between 0 and 0.3 relative intensity. For the C1s core level spectra the X-axis was cut off at the CO_2 gas phase signal, to focus intensity resolution onto surface species.

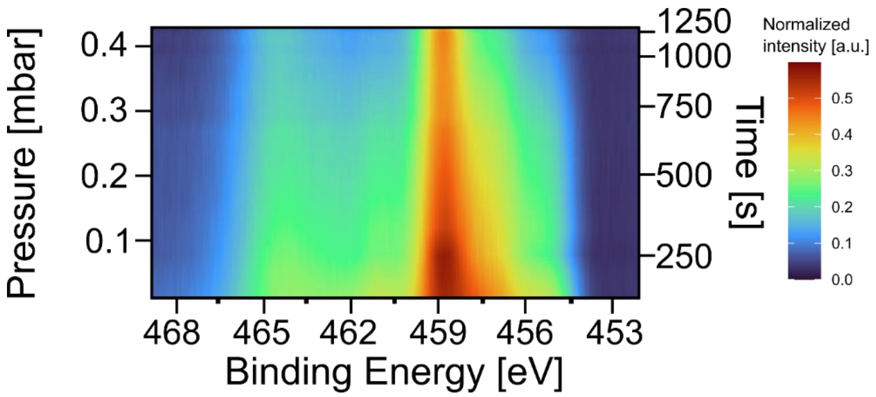

Figure S1 Ti2p AP-XPS core level spectra of highly defective Rutile TiO_2 (110) in different pressures of $CO_{2\,(g)}$. 3D color plot of the Ti2p signal at increasing pressures up to 0.45 mbar CO_2. This figure displays the same data as shown in figure 2a in the main text, but with a different color scale to enhance visibility of the changes in the Ti^{3+} species.

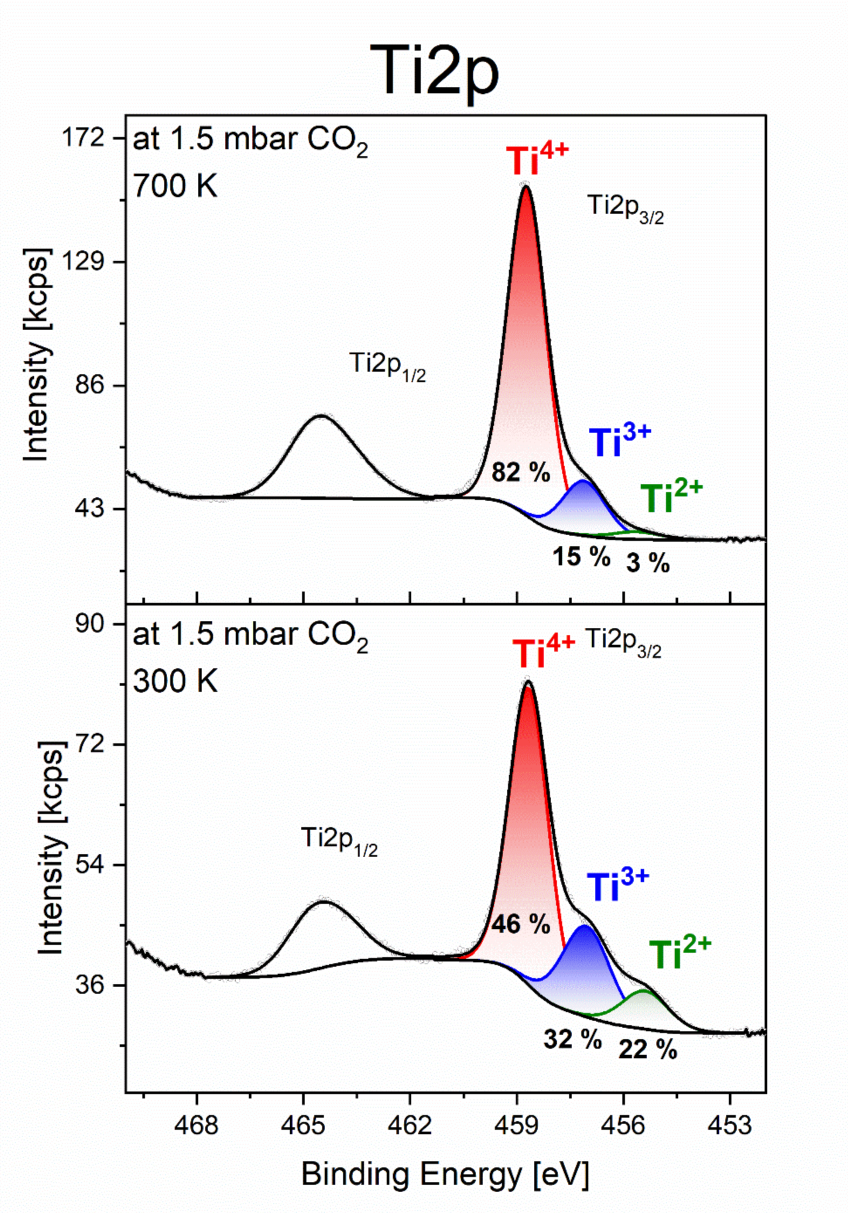

Figure S2 *in-situ* Ti2p AP-XPS core level spectra of highly defective Rutile TiO_2 (110) in 1.5 mbar of $CO_{2\,(g)}$. The measurements were conducted at the Max IV Synchrotron Laboratory. The lower half shows the core level spectrum at 300 K, the upper half shows the core level spectrum at 700 K.

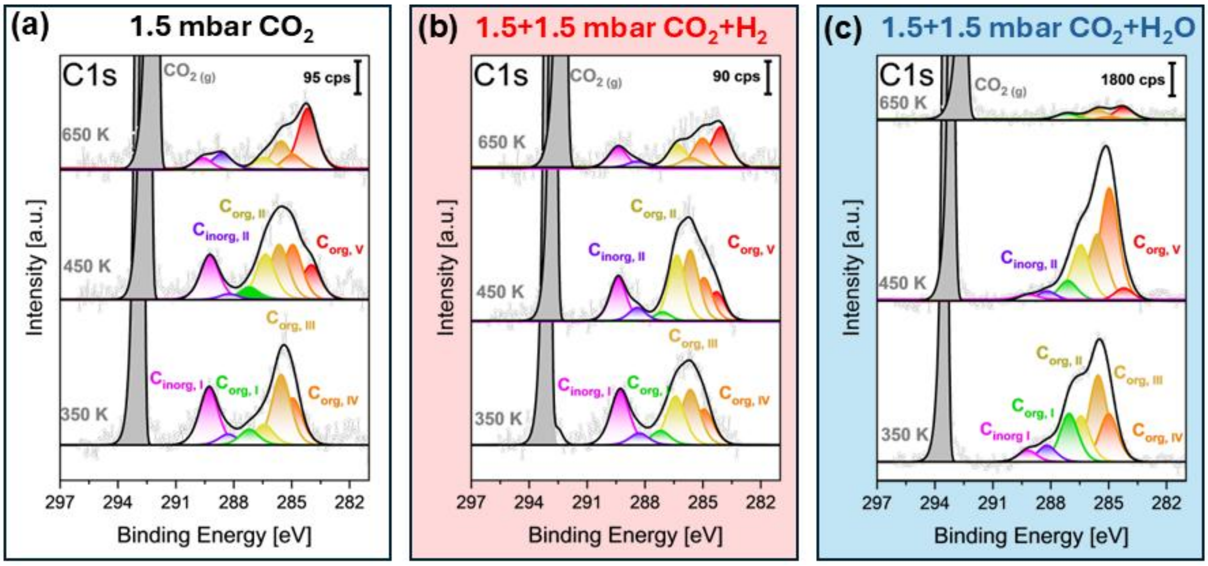

Figure S3 AP-XPS core level spectra of highly defective rutile TiO_2 (110) in different gas mixtures at increasing temperatures. Shown are the C1s core level of (a) 1.5 mbar CO_2, (b) 1.5 mbar CO_2 + 1.5 mbar H_2 and (c) 1.5 mbar CO2 + 1.5 mbar H_2O at increasing temperatures (bottom to top). All C1s spectra are background-subtracted and shifted in height against each other to enable complete investigation of the signal between 282 and 291 eV.

References

1 S. Zhu, M. Scardamaglia, J. Kundsen, R. Sankari, H. Tarawneh, R. Temperton, L. Pickworth, F. Cavalca, C. Wang, H. Tissot, J. Weissenrieder, B. Hagman, J. Gustafson, S. Kaya, F. Lindgren, I. Källquist, J. Maibach, M. Hahlin, V. Boix, T. Gallo, F. Rehman, G. D'Acunto, J. Schnadt and A. Shavorskiy, *J Synchrotron Rad*, 2021, **28**, 624–636.
